# The Eclectic Journals

**Published:** 1885-09-20

**Authors:** 


### THE ECLECTIC JOURNALS.

There comes two or three Eclectic journals to our office. We read them because we are searching in every direction for something to interest and benefit our readers, and, peradventure, we might catch a thought from an Eclectic journal; and yet, if, under like circumstances, we were to exercise the same liberality in the ordinary relations of society, our conduct would be regarded as incompatible with self-respect. It would not be expected of a gentleman to receive visitors into his house who took occasion at every visit to ridicule his sentiments and to heap upon him offensive and opprobious epithets. But this is just what every Eclectic exchange does for us that we receive. Scudder's Journal, which is prominent among that class, is especially given to that sort of exercise toward what he styles *the Regulars.* In his August issue he devotes several pages to this gratifying amusement, bringing in this journal for a share of his courtesies. We often wonder what this class of journals would do if deprived of the privilege of abusing the Regulars; and yet it must be hard to keep it up, as the Regulars rarely notice or respond to their assaults. Usually the most pugnacious animal will lose his ferocity when no opposition is offered to his attacks; but these fellows never tire, from which we infer that the matter of these assaults is not only luxurious to the editors, but ever fresh and attractive to their readers. When they have nothing else to interest their subscribers they can always get up something in this line to fill up with, and feel safe in so doing. This is in keeping with the advice of an old political editor to his son, whom he was instructing in the business of journalism, when he said, "Well, son, when you get out of soap, and can find nothing to say, the best thing to do is to abuse the opposite party."

